# Pituitary Involvement in Granulomatosis with Polyangiitis: A Retrospective Analysis in a Single Chinese Hospital and a Literature Review

**DOI:** 10.1155/2019/2176878

**Published:** 2019-11-06

**Authors:** Shixuan Liu, Yan Xu, Naishi Li, Shi Chen, Shangzhu Zhang, Linyi Peng, Wei Bai, Jinglan Wang, Jinming Gao, Xiaofeng Zeng, Juhong Shi, Mengzhao Wang

**Affiliations:** ^1^Department of Endocrinology, Key Laboratory of Endocrinology of National Health Commission, Peking Union Medical College Hospital, Chinese Academy of Medical Sciences & Peking Union Medical College, Beijing, China; ^2^Department of Respiratory Medicine, Peking Union Medical College Hospital, Chinese Academy of Medical Sciences & Peking Union Medical College, Beijing, China; ^3^Department of Rheumatology, Peking Union Medical College Hospital, Chinese Academy of Medical Sciences & Peking Union Medical College, Beijing, China

## Abstract

**Purpose:**

Granulomatosis with polyangiitis (GPA) is an anti-neutrophil cytoplasmic antibody (ANCA) associated vasculitis that can involve virtually many organs, including the pituitary. Pituitary involvement in GPA is rare, with only case reports or small case series published previously.

**Methods:**

We used the electronic medical record system in our hospital to identify four patients of pituitary involvement in GPA. We summarized the clinical characteristics, radiographic findings, treatments, and clinical outcomes of the four patients. We further performed a systematic literature review of 66 GPA cases with pituitary involvement that were published on the PubMed database.

**Results:**

The four women in our report were between 57 and 73 years of age. All patients had pituitary abnormalities on radiology; three developed diabetes insipidus (DI). All patients had multisystem involvement. After treatment with glucocorticoids and cyclophosphamide (CYC), all patients showed clinical improvement but pituitary function did not resume. Literature review identified 66 additional patients with pituitary involvement in GPA; diabetes insipidus (57/66, 86.4%) and hypogonadism (34/66, 51.5%) were the most frequent pituitary disorders, and the most frequent imaging lesion was an enlarged pituitary (25/64, 39.1%). After treatment with corticosteroids and/or immunosuppressive agents, most patients (45/66, 68.2%) developed remission from systemic disease, 13 patients (13/57, 22.8%) showed remission of DI, and 8 patients (8/46, 17.4%) showed remission of hormone deficiencies.

**Conclusions:**

GPA should be carefully considered as a potential cause of pituitary dysfunction (PD), especially when multisystem dysfunction exists. Conventional treatment with corticosteroids and/or immunosuppressive agents improves systemic symptoms, but pituitary disorders persisted in most patients.

## 1. Introduction

Granulomatosis with polyangiitis (GPA) is an autoimmune small-vessel vasculitis that is strongly associated with anti-neutrophil cytoplasmic antibodies (ANCAs). The incidence of GPA is nearly 10 per million people per year and typically presents between the age of 35 and 55 years, with approximately the same frequency among men and women [1]. Multiple organs can be involved in GPA, including the ear, nose, and throat (ENT); eyes; lungs; kidneys; central nervous system (CNS); and others.

Pituitary involvement is present in approximately 1% of all cases of GPA [2]. To date, only case reports or small case series of pituitary involvement in GPA have been published. Patients with GPA showing pituitary involvement often complain of headache, vomiting, and visual-field defect, suggesting compression of tissues around the pituitary; meanwhile, manifestations of hormone secretion abnormalities, including polyuria, polydipsia, asthenia, amenorrhea, galactorrhea, decreased libido, muscular atrophy, and decreased pilosity, can be observed [3].

GPA should be considered in the differential diagnosis of unexplained diabetes insipidus (DI) with or without partial hypopituitarism, especially when multiorgan involvement existed [4]. Testing for ANCAs, magnetic resonance imaging (MRI) of the pituitary, cerebrospinal fluid (CSF) analysis, and pituitary biopsy are useful to determine the diagnosis. The classic MRI findings are pituitary enlargement, diffuse or focal infundibular thickening, and the absence of the normal high-intensity signal in the posterior pituitary lobe seen on T1-weighted images [5]. CSF analysis mainly serves to exclude other clinical conditions that present with similar signs, such as CNS infection, lymphoma, or Langerhans cell histiocytosis, rather than confirm the diagnosis [6]. The evidence of granulomatous inflammation or inflammatory infiltrates on pituitary biopsy can provide pathological evidence of the clinical diagnosis [3].

Herein, we report on four patients with GPA-related pituitary disease from our Peking Union Medical College Hospital (PUMCH) database and also perform a literature review. We aim to summarize the clinical characteristics, radiographic findings, treatments, and clinical outcomes of patients with pituitary involvement of GPA.

## 2. Patients and Methods

### 2.1. Patients

An electronic medical record system in PUMCH was used to identify patients with GPA-related pituitary disease (PD) from January 1980 to December 2017 by searching the clinical notes. We first searched terms of GPA, Wegener's granulomatosis, and ANCA-associated vasculitis in database to select patients of GPA, and we enrolled 499 patients who fulfilled the diagnostic standards of GPA according to the American College of Rheumatology [7] or the 2012 revised International Chapel Hill Consensus Conference Nomenclature of Vasculitides [8]. Then, we identified four patients with pituitary involvement of GPA using the following terms: GPA, Wegener's granulomatosis, ANCA-associated vasculitis, pituitary disease, pituitary dysfunction, pituitary insufficiency, pituitary abnormality, pituitary tumor, pituitary enlargement, or DI.

This study was approved by the PUMCH Ethics Committee and followed the ethical standards of the responsible committee on human experimentation (institution and national) and with the Helsinki Declaration of 1964, as revised in 2013. Informed consent for publication of the clinical information including laboratory examinations, clinical images, and so on was obtained from each patient at the time of diagnosis or follow-up.

### 2.2. Medical Information

Medical information was collected, including information on the diagnostic evidence of GPA, evaluation of pituitary function, treatment, and clinical outcome. Data included those on the onset of disease, clinical symptoms, organs involvement, tissue biopsies, radiological findings, and ANCA titers; additionally, data on the levels of inflammatory markers, such as hypersensitive C-reactive protein (hsCRP), and the erythrocyte sedimentation rate (ESR) were collected to assess the state of GPA. Pituitary involvement was based on the anterior and posterior pituitary hormone levels and typical lesions on imaging of the pituitary.

### 2.3. Evaluation of the Pituitary

The assessment of pituitary hormone function was classified by five axes for the anterior pituitary (gonadotropin axis, thyrotropin axis, growth hormone axis, corticotropin axis, and prolactin) and one for the posterior pituitary.

Gonadotropin insufficiency was confirmed by low levels of serum gonadal steroid including follicle-stimulating hormone and luteinizing hormone with or without deficiency of gonadotropin containing estradiol and testosterone. Hypothyroidism was defined as low free thyroxine without thyroid-stimulating hormone appropriate elevation, thyrotropin releasing hormone stimulation test was necessary in distinguishing pituitary or hypothalamic origin while not being able to carry out in Chinese hospital at present. Growth hormone (GH) insufficiency was suspected on the basis of abnormal low insulin-like growth factor-1 (IGF-1). Adrenal insufficiency was diagnosed on the basis of low morning cortisol (<5 *μ*g/dL) with low or inappropriately normal adrenocorticotropic hormone levels. As some patients had already received corticosteroid treatment, the adrenal axis was not evaluated. Hyperprolactinemia was diagnosed on the basis of a high level of serum prolactin.

DI was diagnosed by combining the clinical symptoms of polyuria and polydipsia with laboratory tests demonstrating dilute urine and simultaneous hypotonic urine with osmotic pressure inappropriately lower than that of plasma. Water deprivation-vasopressin test was a confirmatory diagnostic test but was not performed in all patients since they have already received replacement treatment of desmopressin before coming to our hospital, and they all responded well to desmopressin treatment.

### 2.4. Literature Review

We searched PubMed using the terms “ANCA-associated vasculitis” or “vasculitis” or “Wegener's granulomatosis” or “granulomatosis with polyangiitis” and “hypophysitis” or “pituitary tumor” or “pituitary dysfunction” or “pituitary disease” or “diabetes insipidus” to identify all articles in English. To date, 2 large case series [2, 3] and 35 small case series or case reports [9–43] of pituitary involvement in GPA had been published.

### 2.5. Data Collection and Statistical Analysis

The descriptive results were presented as percentages or ranges. Two authors extracted the medical information of the enrolled patients and the literatures using standardized forms. Data from literatures were primarily descriptive. All statistical analyses were performed using SPSS 22.0 software (IBM-SPSS Inc., Chicago, IL, USA).

## 3. Results

### 3.1. Our Patients

**Figure 1:**
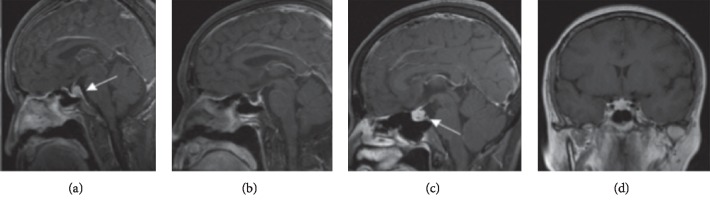
Dynamic magnetic resonance imaging (MRI) of the pituitary (T1-weighted). Enlargement of the posterior pituitary lobe and absence of the hyperintense signal of the posterior pituitary gland on T1-weighted images of patient No. 1 (a). Reappearance of posterior pituitary bright spot and the mass of the posterior pituitary on T1-weighted images of the same patient almost completely resolved after treatment (b). Enlargement of a space-occupying lesion and loss of the normal posterior pituitary T1 hyperintense signal on dynamic MRI of the pituitary (T1-weighted) of patient No. 2 (c, d).

In this study, we described four patients with pituitary involvement by GPA (4/499, 0.8%). Three patients developed DI, and two patients presented with DI as the initial symptom leading to the diagnosis of GPA. In our patients, endocrine involvement included DI, mild hyperprolactinemia, and hypothyroidism. However, the fact that prolactin is a kind of stress hormone and the elevation of prolactin was just mild in our two patients may not suggest a morbid state. All patients had multisystem involvement, including ENT (4/4, 100%), lung (2/4, 50%), kidney (2/4, 50%), eyes (2/4, 50%), CNS (1/4, 25%), and peripheral nervous system (1/4, 25%). All patients had abnormal pituitary images on MRI and loss of the posterior hypersignal (Figures 1(a), 1(c), and 1(d)). Concerning serology, perinuclear ANCA (p-ANCA) was positive in all patients with positive myeloperoxidase (MPO). One patient underwent pulmonary biopsy; pathologic examination revealed inflammatory exudation. All patients responded well to glucocorticoid and cyclophosphamide (CYC) treatment, with improvement of systemic disease but persistent DI. The anterior pituitary function was not reassessed in our patients. There was only one patient who underwent repeat MRI after treatment, which revealed a nearly normalized pituitary and reappearance of the lost posterior signal (Figure 1(b)). After treatment, the MPO-ANCA level in all patients declined to negative.  Table 1 summarizes the clinical characteristics, radiological findings, treatment, and outcome of our four cases.

### 3.2. Literature Review

#### 3.2.1. Patient Characteristics

In total, 66 patients with a confirmed diagnosis of GPA-related pituitary disease were enrolled in the literature review with a female predominance (69.7%).

#### 3.2.2. Serology

The reported typical ANCA pattern associated with GPA is cytoplasmic ANCA (c-ANCA) recognizing proteinase 3 (PR3). Of the 59 cases in which ANCA results were reported, 55 were positive, with 3 patients confirmed positive for p-ANCA with MPO and 50 positive for c-ANCA (with 37 confirmed positive for PR3). Especially, one patient whose ANCA in serum was negative proved MPO-positive neutrophils in pituitary pathology, which was thought to be consistent with pituitary involvement in GPA due to multiple lineages of inflammatory cells and MPO positivity [30].

#### 3.2.3. Pituitary Function and Other Organ Involvement

**Figure 2:**
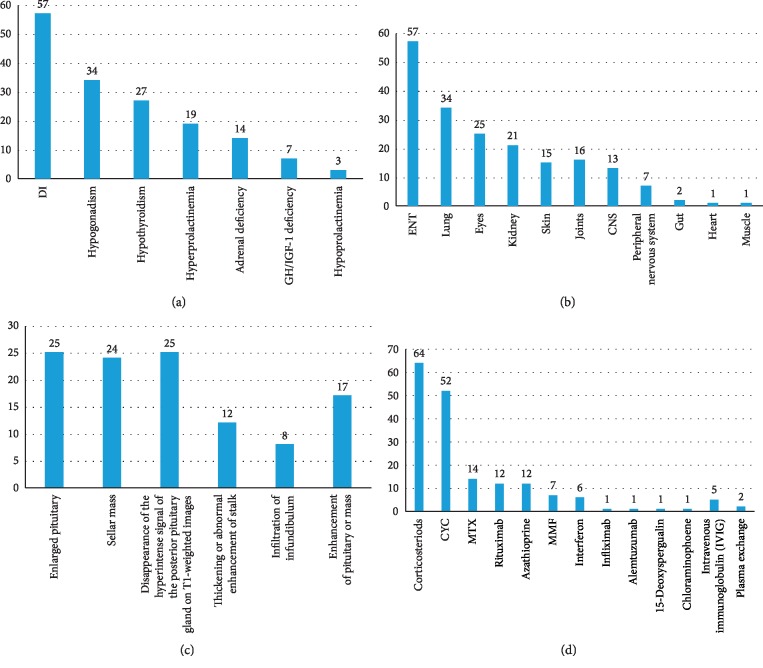
(a) Manifestations of pituitary dysfunction of the literature review. (b) Other organ involvement of the literature review. (c) Radiological abnormalities of the pituitary of the literature review. (d) Treatments of GPA of the literature review.

There were 57 patients who developed DI and 46 patients with anterior pituitary hormone deficiencies reported during the course of GPA, with 23 patients presenting DI as the initial feature. Anterior PD alone was reported in 9 patients and posterior PD alone in 13 patients; 37 patients had both anterior and posterior PD, and 7 patients did not have assessment of anterior pituitary function. Other organs involved in GPA included ENT, lung, eyes, kidney, skin, joints, nervous system, and so on. The results of pituitary dysfunction and multiorgan involvement are separately summarized in Figures 2(a) and 2(b).

#### 3.2.4. Radiological Findings

Pituitary MRIs were evaluated in 64 patients, among whom 60 showed abnormalities and the findings were summarized in Figure 2(c).

#### 3.2.5. Treatment

Treatments administered in all the cases were diverse (Figure 2(d)). All patients received corticosteroids (64/66, 97.0%) and/or immunosuppressive agents (62/66, 93.9%).

Supplementary [Supplementary-material supplementary-material-1] summarizes specific data of clinical characteristics, radiological findings, and treatment of previously reported cases of pituitary involvement in GPA.

#### 3.2.6. Clinical Outcome

After treatment, most patients recovered from systemic disease; however, most patients remained persistent DI and so do the results of hormone deficiencies. A subsequent MRI was available in 47 cases and the loss of posterior signal was reevaluated in 8 patients. Two patients died—1 of these patients developed remission but was diagnosed with overwhelming sepsis 13 months after the initial diagnosis.  Table 2 summarizes follow-up of patients with GPA-related pituitary disease.

Supplementary [Supplementary-material supplementary-material-1] shows the initial date of previously reported cases of follow-up of patients with GPA-related pituitary disease.

## 4. Discussion

In this study, we described 4 cases of PD involvement in GPA from a single center, and summarized patient characteristics, clinical presentations, radiology findings, treatments, and general prognosis. We also performed, to the best of our knowledge, the largest case review to date of pituitary involvement in GPA, for a systemic investigation of this cohort of patients. We revealed several interesting findings from our study and the literature review: (1) the prevalence of pituitary disease in our cohort was 0.8%, which was in agreement with the findings of previous research; (2) hypogonadism and DI were the most frequent pituitary disorders in patient with GPA; (3) corticosteroids and/or immunosuppressive therapy was the standard treatment for those patients; and (4) most patients (4/4 [100%] in our study, 45/66 [68.2%] in the literature review) showed remission from systemic disease at the end of follow-up, whereas the outcome of pituitary function was less favorable.

Nervous system involvement is not uncommon in GPA, with a reported frequency ranging from 22% to 54% among patients; CNS involvement is observed in only 7%–11% of patients with GPA [3]. First reported in 1953 [44], pituitary involvement in GPA is rare, representing approximately 1% of patients in several large cohorts of GPA [1, 9, 45]. The exact pathophysiological mechanism is still elusive and there are three major theories [9]. The first is by direct extension of the granulomatous lesion from contiguous sites including the ENT and the orbits, the second is through cerebral vascular involvement, and the last is the direct formation of granuloma at the affected site.

Kapoor et al. [2] reported 8 well-documented cases of PD among 637 patients with GPA (1.3%) recruited in a tertiary referral center between 1996 and 2011; the average age of patients with confirmed pituitary involved was 48 years (range: 28–67 years) with no gender difference. Another study reported by De Parisot et al. [3] included 9 patients with pituitary involvement among 819 patients with GPA (1.1%) in the French Vasculitis Study Group database; the median age at diagnosis of PD was 50.8 years (range: 24–77 years), with an almost 1 : 1 male-to-female ratio. The low prevalence of pituitary disease in our cohort (0.8%) was in agreement with that in the previously published research. In contrast, there was an absolute female predominance. However, it was certainly likely that a significant proportion of patients missed or received a delayed diagnosis since the early clinical features of pituitary involvement, such as fatigue, headache, dizziness, and visual change, are nonspecific and easily ignored.

ANCAs are valuable laboratory markers used for the diagnosis of ANCA-associated vasculitis (AAV). Two major immunostaining patterns can be seen with the indirect immunofluorescence assay (IIF): c-ANCA and p-ANCA [46]. Enzyme-linked immunosorbent assay (ELISA) is quantitative method to measure PR3-ANCA and MPO-ANCA titers. The use of IIF and ELISA in ANCA testing gives a 96% sensitivity and 98.5% specificity for AAV [47], with 80% of patients with GPA being seropositive for PR3-ANCA and 10% for MPO-ANCA in Western countries [48]. However, in Chinese patients with systemic GPA, 60%–70% of them were p-ANCA specificity of MPO [49, 50]. In the literature review, almost all patients testing for ANCA were PR3-positive except for three; however, our four patients were all positive for MPO-ANCA. Positive ANCA is one of the diagnostic criteria for GPA, but the diagnosis cannot be excluded in patients who are ANCA-negative. Recently, related publications have pointed out that the likelihood of GPA increases with increasing ANCA levels [51], but ANCA levels do not always correlate with disease severity [5]. As for the significance of ANCAs to evaluate the prognosis of the disease, there is no explicit conclusion. We observed that ANCA titers fell gradually to a low titer in our four patients, coinciding with systemic disease remission.

Hypogonadism and DI were the most frequent pituitary disorders reported in our study, in agreement with the reports of Kapoor et al. (hypogonadism, 87.5%; DI, 75%) [2] and De Parisot et al. (hypogonadism, 78%; DI, 78%) [3]. Based on our review, the frequency of DI in patients with pituitary involvement was 85.7% (60/70) with 35.7% (25/70) of patients having this finding as the presenting feature. Gonadotropin deficiency affected 48.6% (34/70) of patients. Nevertheless, the involvement of hypogonadism in the pituitary GPA might be overestimated as a result of suppression of the hypothalamic-pituitary-gonadal axis due to treatment with glucocorticoids and CYC.

Other hormone deficiencies in GPA-related PD were not as common as DI and hypogonadism, including hypothyroidism (28/70, 40.0%), hyperprolactinemia (21/70, 30.0%), adrenal deficiency (14/70, 20.0%), and GH/IGF-1 deficiency (7/70, 10.0%).

Pituitary MRI is of significant importance in confirming pituitary involvement in GPA. The most common finding is pituitary enlargement or adenoma, described in 80% of patients [2]. Other classic radiology lesions in GPA are diffuse or focal infundibular thickening and the absence of the posterior hypersignal on T1-weighted images. MRI abnormalities were present in 94.1% (64/68) of patients, with the MRI information of two patients lacking in the literature review. In agreement with previous reports, pituitary enlargement or pituitary mass were the most frequent lesions, being present in 77.9% (53/68) of patients, followed by disappearance of the posterior hypersignal on T1-weighted images in 42.6% (29/68), thickening or abnormal enhancement of the stalk in 17.6% (12/68), and infiltration of infundibulum in 11.8% (8/68).

With the current limitations of noninvasive tests, tissue biopsy is still necessary for confirmation of the diagnosis because of the potentially toxic nature of the treatments available for GPA. However, pituitary biopsy is currently not a primary recommendation, given the excellent response to treatment of glucocorticoids combined with immunosuppressive agents and the potential risks of the invasive biopsy procedure. Meanwhile, most patients with pituitary involvement in GPA show other organ involvement, providing the possibility of performing tissue biopsy at other organs. In our review, there were 48 patients in whom histological evidence was obtained, including 11 from the pituitary, 28 from other organs involved in GPA including kidney, lung, skin, nasopharyngeal, and nerve tissue, and exact location was not reported for other patients. None of our patients underwent pituitary biopsy; one underwent pulmonary tissue biopsy, which showed an inflammatory exudation. Our patients preferred to try conventional treatment for GPA first, and to consider pituitary biopsy after evaluating the treatment outcome. However, biopsy or surgery is still necessary for those patients with pituitary disease that progresses during regular treatment or those that show relapse.

Currently, almost all patients with PD due to GPA are treated with the standard therapy used for severe GPA, consisting of high-dose glucocorticoids combined with oral or intravenous CYC; remission is reported in two-thirds of patients [3, 25]. In a previous retrospective review of 23 patients with pituitary involvement by GPA reported in the English literature between 1966 and 2006, 65% of patients were treated with a CYC-based regimen, with a relapse rate of 27% occurring at a median of 10.5 months (range: 7–36 month) versus a relapse rate of 50% at a median of 4.5 months (range: 4–18 month) without CYC [15]. In our literature review, 78.6% (55/70) of patients were treated with this traditional therapy, whereas others substituted other kind of immunosuppressant for CYC; one patient was treated with immunosuppressant only. There were four patients treated with high-dose glucocorticoids alone to achieve disease remission, but one of these patients began CYC to control disease relapse during the process of decreasing the glucocorticoid dose. It is important to note the fact that high-dose glucocorticoids have a high rate of side effects without improving treatment efficacy in many immunologic diseases, and high-dose glucocorticoids, rather than immunosuppression, are now considered to increase the rate of severe infection [52]. In newly diagnosed and severe relapsing GPA, RTX in combination with glucocorticoids has been approved by the U.S. Food and Drug Administration based on data from the Rituximab in ANCA-Associated Vasculitis trial (RAVE) [53]. However, there is limited experience with the use of RTX in pituitary involved GPA, and the response of pituitary GPA treated with RTX deserves further study. With more severe disease, especially pulmonary hemorrhage or a rapidly progressive renal failure, plasma exchange is a supplementary strategy targeted towards removing the offending antibodies. Observational work also suggests that those with highly active ANCA-associated vasculitis are more likely to benefit from plasma exchange than those with a chronic presentation [52].

Despite a high rate of systemic disease remission of 70.0% (49/70), the outcome of pituitary function was less favorable. The rate of anterior and posterior pituitary functional recovery was 16.7% and 21.7% (8/48 for hormone disabilities and 13/60 for DI) in our cohort and literature review cases. The high rate of residual pituitary disability was in agreement with the studies reported by De Parisot et al. and Kapoor et al. [2, 3], where 86% and 62.5% of patients, respectively, did not show recovery of pituitary function, probably due to permanent pituitary damage related to necrotizing granulomatous inflammation of the gland [3]. After treatment, it reported that 35.4% (17/48) of all cases showed radiological resolution, and 39.6% (19/48) of patients showed reduction in the size of the pituitary lesions on MRI. No correlation was found between pituitary function and radiologic and general outcomes, as confirmed in our study and by two other investigations [2, 3]. It is thus necessary to assess both imaging findings and pituitary function to evaluate the total treatment response.

There are several limitations in our study. First, it was a retrospective study, and there were a limited number of cases. The second limitation of this article was that all patients in our study did not undergo biopsy to obtain histological evidence of GPA. The third limitation was that three patients in our case series did not have imaging data during follow-up. The systemic literature review can give us some additional information, to complement limitations of our own case series.

## 5. Conclusion

GPA is a multisystem disease characterized by necrotizing small-vessel vasculitis that can involve the pituitary, and PD can develop at any time during the course of the disease. GPA should be carefully considered in patients with pituitary lesions on radiological imaging or presenting with symptoms of DI or anterior pituitary dysfunction. Although conventional treatment improves systemic symptoms, abnormalities of both pituitary function and radiologic findings persist in most patients. We should bear in mind that GPA can be the cause of PD, and systematic therapy should be initiated at an early stage to induce remission of systemic disease and improved pituitary function.

## Figures and Tables

**Table 1:** Characteristics, ANCA results, pituitary function, radiographic findings, treatment, and outcome of patients with GPA-related pituitary disease.

Case no.	1	2	3	4
Age (y)	64	62	73	57
Sex	F	F	F	F
ANCA testing	p-ANCA and MPO	p-ANCA and MPO	p-ANCA and MPO	p-ANCA and MPO
PD as the presenting feature	Yes	No	Yes	No
DI	Yes	Yes	Yes	No
Anterior pituitary function	Mild hyperprolactinemia	Normal	Normal	Mild hyperprolactinemia, hypothyroidism
Other organ involvement	ENT, lung	ENT, lung, eyes, true bulbar palsy	ENT, kidney	ENT, eyes, kidney, CNS (hypophysitis, hypertrophic spinal pachymeningitis, pachymeningitis, and myelitis)
Radiology findings	Thickening of posterior pituitary lobe and absence of the hyperintense signal of the posterior pituitary gland on T1-weighted images	Enlargement of the pituitary and loss of the normal posterior pituitary T1 hyperintensity	Posterior pituitary mass with loss of the hyperintense signal of the posterior pituitary gland on T1-weighted images	Pituitary enlargement with decreased local enhancement and loss of the hyperintense signal of the posterior pituitary gland on T1-weighted images
Treatment	GC, CYC (IV and oral)	GC, oral CYC	GC, CYC	GC, IV CYC, triptolide, IVIG
Pituitary imaging post treatment	Improved	Not reported	Not reported	Not reported
Pituitary function post treatment	Persistent DI	Persistent DI	Persistent DI	Not reported
Systemic disease post treatment	Improved	Improved	Improved	Improved

PD = pituitary dysfunction, DI = diabetes insipidus, F = female, p-ANCA = perinuclear anti-neutrophil cytoplasmic antibody, MPO = myeloperoxidase, ENT = ear, nose, and throat, CNS = central nervous system, GC = glucocorticoid, CYC = cyclophosphamide, IVIG = intravenous immunoglobulin, IV = intravenous.

**Table 2:** Follow-up of patients with GPA-related pituitary disease.

Patient characteristic	*N*
*Pituitary imaging*	
No change	11
Normalized	17
Reduction	18
Enlargement	1
Reappearance of posterior signal	0
Persistent loss of posterior signal	8
Total	47

*Anterior pituitary function*	
Remission	8
Persistent insufficiency	23
Partial remission	2
Not reported	12
Death	1
Total	46

*Posterior pituitary function*	
Remission	13
Persistent insufficiency	32
Partial remission	2
Not reported	8
Death	2
Total	57

*Systemic disease*	
Remission	39
Relapse then stabilized	6
Relapse then remission	6
Relapse then no information	2
Stabilized	2
Progression	1
Death	2
Not reported	8
Total	66

*N* = number.

## Supplementary Materials

Supplementary Materials Supplementary Table 1: characteristics, ANCA results, pituitary function, radiographic findings, treatment, and outcome of patients with GPA-related pituitary disease. Supplementary Table 2: raw follow-up of patients with GPA-related pituitary disease.
