# Supplementary material for: Pituitary Involvement in Granulomatosis with Polyangiitis: A Retrospective Analysis in a Single Chinese Hospital and a Literature Review
Source: Int J Endocrinol. 2019 Nov 6;2019:2176878. doi: 10.1155/2019/2176878 (PMC6874975; doi:10.1155/2019/2176878)
Supplement: Supplementary Materials — Supplementary Table 1: characteristics, ANCA results, pituitary function, radiographic findings, treatment, and outcome of patients with GPA-related pituitary disease. Supplementary Table 2: raw follow-up of patients with GPA-related pituitary disease. [file 2176878.f1.zip › 2176878.f1/Supplementary Table 1.docx]

| **Supplementary Table 1. Characteristics, ANCA Results, Pituitary Function, Radiographic Findings, Treatment, and Outcome of Patients with GPA-Related Pituitary Disease** | | | | | | | | | | | |
| --- | --- | --- | --- | --- | --- | --- | --- | --- | --- | --- | --- |
| **Case  No** | **Age， y** | **Sex** | **ANCA testing** | **PD as the  presenting feature** | **DI** | **Anterior pituitary function** | **Other organ involvement** | **Radiology findings** | **Treatment** | | **Reference** |
| 1 | 67 | F | all but one tested positive for ANCA reacting with PR3 | Yes for four | No | Mild hyperprolactinemia, Hypogonadism | ENT, Kidney | 11-mm peripherally enhancing cystic sellar mass compressing the stalk | GC+CYC | | 2 |
| 2 | 48 | F |  |  | Yes | Hypothyroidism,  Adrenal insufficiency, Hypoprolactinemia,  Hypogonadism | ENT, Lung, Skin | Multiple non-enhancing cystic areas in the pituitary, convexity of superior margin of pituitary gland | GC+CYC | | 2 |
| 3 | 28 | F |  |  | Yes | Hypogonadism | ENT, Lung, Kidney | 15-mm sellar mass with large zone of central non-enhancement and peripheral enhancement, stalk preserved on initial imaging, but later displaced posteriorly | GC+CYC | | 2 |
| 4 | 55 | M |  |  | Yes | Hypogonadism | ENT, Lung, Kidney, Skin, Joints | 10-mm sellar mass with suprasellar extension | GC+CYC | | 2 |
| 5 | 35 | M |  |  | Yes | Hypothyroidism,  Hypogonadism | ENT | 15-mm necrotic sellar mass with peripheral enhancement and suprasellar extension, thickening and abnormal enhancement of the stalk, loss of posterior signal | GC+CYC | | 2 |
| 6 | 54 | M |  |  | Yes | Hypothyroidism,  Hypogonadism | ENT, Lung, Kidney, Heart | Enlarged pituitary (12-mm) with abnormal heterogeneous enh  ancement, slight diffuse thickening of the stalk | GC, CYC, Rituximab | | 2 |
| 7 | 68 | M |  |  | No | Hypothyroidism,  Hypoprolactinemia,  Hypogonadism,  IGF-1 deficiency | ENT, Joints | 16-mm homogeneously enhancing sellar mass, extending into the cavernous sinus bilaterally | GC, Rituximab | | 2 |
| 8 | 28 | F |  |  | Yes | Normal | ENT | 13-mm sellar mass extending into the suprasellar cistern, with low T2 signal in  the periphery and a bright center, peripheral enhancement with central cystic  change, thickening of pituitary stalk | GC, Rituximab | | 2 |
| 9 | 46 | F | 7 were ANCA-positive, of whom 6 were PR3- ANCA-positive | Yes | Yes | Not reported | ENT, Eyes | Enlarged posterior pituitary with infiltration, loss of posterior signal | GC, MTX, IV CYC | | 3 |
| 10 | 60 | M |  | No | No | Hypogonadism,  Hypothyroidism,  GH deficiency | ENT, Peripheral neuropathy, Cranial nerve palsy | Normal | GC, Rituximab, Chloraminophene, IVIG | | 3 |
| 11 | 23 | F |  | No | Yes | Hypogonadism,  Hypothyroidism | ENT | Enlarged pituitary, irregularity of infundibulum, heterogeneous enhancement of anterior pituitary, loss of posterior signal | GC, MTX 20 mg, INF 3 mg/kg, Rituximab | | 3 |
| 12 | 24 | M |  | No | Yes | Hypogonadism,  Hypothyroidism,  Hyperprolactinemia | Kidney, Gut, Joints, Muscle, Eyes | Enlarged infundibulum, loss of posterior signal | GC, MTX 15 mg subcutaneous, IVIG | | 3 |
| 13 | 66 | M |  | No | No | Hypogonadism,  Hypothyroidism,  GH deficiency | No | Enlarged pituitary, loss of posterior signal,  infiltration of infundibulum | GC | | 3 |
| 14 | 67 | F |  | No | Yes | Hyperprolactinemia | ENT, CNS | Normal | GC+IV CYC | | 3 |
| 15 | 28 | F |  | No | Yes | Hypogonadism,  Hyperprolactinemia,  Adrenal insufficiency | ENT, Lung | Heterogeneous enhancement  of pituitary | GC, MTX, INF, MMF | | 3 |
| 16 | 55 | M |  | No | Yes | Hypogonadism,  Hypothyroidism,  Hyperprolactinemia,  Adrenal insufficiency | CNS, Lung | Sellar mass, heterogeneous enhancement, enlargement and infiltration of infundibulum, loss of posterior signal | GC, Oral CYC 200 mg, AZA | | 3 |
| 17 | 46 | F |  | No | Yes | Hypogonadism,  Hyperprolactinemia | ENT, Eyes | Enlargement and infiltration of pituitary with heterogeneous enhancement, contact with optic chiasm | INF 5 mg/kg, AZA | | 3 |
| 18 | 33 | F | c-ANCA and PR3 | Yes | Yes | Hypothyroidism,  Adrenal insufficiency,  Hyperprolactinemia,  Hypogonadism | ENT, Eyes | Pituitary mass,  optic chiasm compression | GC,  Oral CYC 100 mg daily,  AZA 100 mg daily | | 4 |
| 19 | 61 | F | c-ANCA and PR3 | Yes | Yes | Not reported | Kidney, Lung | Loss of posterior signal | GC,  Oral CYC 50 mg daily for 6 months,  AZA 100 mg daily | | 4 |
| 20 | 37 | F | c-ANCA and PR3 | No | Yes | Hypogonadism,  GH deficiency | ENT | Sellar mass | GC, CYC, MTX | | 9 |
| 21 | 36 | F | c-ANCA and PR3 | Yes | Yes | Normal | ENT, Lung | Cystic pituitary mass, loss of posterior signal | GC, CYC,  Plasma exchange | | 9 |
| 22 | 32 | F | c-ANCA and PR3 | No | Yes | Normal | ENT | Sellar mass, thickening of the pituitary stalk, mild compression of optic chiasm | GC, CYC, AZA | | 9 |
| 23 | 56 | M | c-ANCA and PR3 | No | Yes | Normal | ENT, Facial nerve palsy | Pituitary macroadenoma | GC, IV CYC, MTX, Rituximab | | 10 |
| 24 | 40 | F | Not reported | No | Yes | Hypothyroidism,  Hyperprolactinemia,  Hypogonadism | ENT, Kidney, Lung | Enlargement and avid contrast enhancement of the pituitary stalk and gland, loss of posterior signal | GC, CYC, Rituximab | | 11 |
| 25 | 53 | F | c-ANCA | No | Yes | Hypothyroidism,  Hypogonadism,  Mild hyperprolactinemia | ENT, Kidney, Lung, Skin | Loss of posterior signal, discrete thickening of the pituitary stalk | GC+CYC | | 12 |
| 26 | 26 | M | c-ANCA and PR3 | Yes | Yes | Not reported | ENT, Lung, Skin | Not reported | GC, IV CYC monthly,  AZA | | 13 |
| 27 | 38 | F | c-ANCA | No | No | Hypothyroidism,  Hypogonadism | ENT, Kidney, Lung, CNS | Infundibular thickening and a 22 × 14 × 14 mm sellar mass with hypointensity and loss of the posterior signal | GC | | 14 |
| 28 | 33 | M | c-ANCA and PR3 | Yes | Yes | Adrenal insufficiency,  Hypogonadism | ENT, Eyes | Thickening of the pituitary stalk and loss of hyperintense signal of posterior signal | GC+Oral CYC | | 15 |
| 29 | 37 | F | negative→c-ANCA | Yes | Yes | Normal | ENT, Lung, Eyes, Skin, Joints | Loss of posterior signal | GC, IV CYC 1000 mg for 6 cycles, Oral CYC 100 mg daily | | 16 |
| 30 | 40 | F | c-ANCA and PR3 | Yes | Yes | Not reported | ENT, Lung | Mildly enlarged pituitary gland with loss of posterior signal | GC, IV CYC,  AZA 50mg twice daily | | 16 |
| 31 | 47 | F | Not reported | No | Yes | Normal | ENT, Kidney, Lung, Joints, Eyes | Not reported | GC+CYC | | 17 |
| 32 | 13 | M | ANCA | Yes | Yes | Normal | ENT, Lung | Mild diffuse enlargement of the  pituitary gland (12-mm height),  loss of posterior signal | GC+IV CYC | | 18 |
| 33 | 47 | F | c-ANCA and PR3 | Yes | Yes | Hypothyroidism,  Adrenal insufficiency,  Hypoprolactinemia, Hypogonadism | ENT, Lung, Skin | Cystic enlargement of the pituitary gland | GC+CYC | | 19 |
| 34 | 48 | F | c-ANCA | No | Yes | Mild hypothyroidism | ENT, Kidney, Lung, Eyes | Sellar mass, heterogeneous enhancement of the pituitary | GC | | 20 |
| 35 | 21 | F | Not reported | No | Yes | Not reported | ENT, Lung, Eyes, Joints | Normal | GC,  MTX 7.5 mg | | 21 |
| 36 | 71 | F | c-ANCA | No | Yes | Hypothyroidism,  Adrenal insufficiency,  Hypogonadism | Eyes | Intrasellar mass | GC at first,  oral CYC after relapse | | 22 |
| 37 | 28 | F | c-ANCA | No | Yes | Partial GH deficiency | ENT, Eyes, Kidney, Skin, Partial third nerve palsy | Intrasellar mass | GC, IV CYC, IVIG | | 22 |
| 38 | 34 | F | Not reported | No | Yes | Hyperprolactinemia | ENT | Enlargement of the sellar mass accompanied by enlargement of the infundibulum | GC | | 23 |
| 39 | 36 | F | ANCA were absent at 2 and 5 months after illness onset. | No | Yes | Hypothyroidism,  Hypogonadism | ENT, Lung, CNS, Eyes | Macrocystic pituitary tumor | GC+IV CYC | | 24 |
| 40 | 41 | F | c-ANCA | Yes | Yes | Hyperprolactinemia | ENT, Joints, Skin, Eyes | Pituitary enlargement,  loss of posterior signal | GC,  MTX 15 mg weekly | | 25 |
| 41 | 18 | F | c-ANCA | Yes | Yes | Hyperprolactinemia | ENT, Lung | Enlarged pituitary gland (14-mm height) and the loss of posterior signal | GC, MTX 15 mg weekly and then CYC 150 mg daily | | 25 |
| 42 | 45 | M | c-ANCA with PR3 and MPO | Yes | Yes | Hyperprolactinemia, Hypogonadism | ENT, Kidney, Lung, Eyes, CNS (polyneuropathy and hemicerebellar syndrome) | Infundibular thickening and the loss of posterior signal | GC+Oral CYC 2 mg/kg | | 26 |
| 43 | 47 | F | c-ANCA and PR3 | No | Yes | Normal | ENT, Kidney, Lung, Joints, Eyes | Loss of posterior signal | GC+CYC 500 mg/m2 for 6 bolus | | 27 |
| 44 | 50 | F | c-ANCA and PR3 | No | No | Hypothyroidism,  Adrenal insufficiency,  Hypogonadism,  Hyperprolactinemia | ENT, Joints, Skin, Eyes | Enlarged pituitary, loss of posterior signal | MTX 15 mg weekly→  IV CYC 600 mg/m2 every 3 weeks for 9 months→oral CYC 1.5 mg/kg daily+GC;  AZA 2 mg/kg daily, INF 5 mg/kg, GC 5 mg daily | | 28 |
| 45 | 41 | F | c-ANCA and PR3 | No | Yes | Normal | ENT, Kidney, Joints, Eyes | Nodular enlargement and enhancement  of pituitary | Oral (1.5 mg/kg daily) and IV (0.6 mg/m2 every 3 week) CYC→AZA 1.5 mg/kg daily→INF 5 mg/kg, MTX 15 mg weekly, MMF 2 g daily; GC 1 mg/kg daily→10 mg daily | | 28 |
| 46 | 57 | M | c-ANCA and PR3 | No | Yes | Panhypopituitarism,  Hyperprolactinemia | ENT, Kidney, Lung, Eyes, Joints, CNS (TIA of the middle cerebral artery, meningitis), Peripheral polyneuropathy | Enlargement of the pituitary gland (13-mm in diameter), with central necrosis and the loss of posterior signal | GC and IV CYC; 15-deoxyspergualin; maintained with AZA 2 mg/kg daily | | 28 |
| 47 | 57 | F | c-ANCA and PR3 | Yes | Yes | Not reported | ENT, Kidney, Lung, Skin | Pituitary mass with  low intensity center | GC, CYC 50 mg,  plasma exchange | | 29 |
| 48 | 30 | F | Negative | No | Yes | Hypothyroidism,  Adrenal insufficiency,  Hypogonadism | CNS, Eyes, Skin | Enhancing sellar mass with compression of the optic chiasm and cavernous sinus | GC, CYC, MTX, Infliximab, MMF, Rituximab | | 30 |
| 49 | 48 | F | Not reported | No | No | Hypothyroidism,  Hyperprolactinemia | ENT, Eyes | Heterogeneous pituitary gland, diffusely enlarged pituitary gland with cystic component, mildly enlarged sellar, thickened pituitary stalk,  compression of optic chiasm | GC, CYC; MMF | | 31 |
| 50 | 37 | F | c-ANCA | No | Yes | Hyperprolactinemia | ENT, Lung, Joints | Loss of posterior signal, enlarged anterior pituitary,  peripheral enhancement of the gland, infundibular enhancement | GC+CYC | | 32 |
| 51 | 63 | F | c-ANCA and PR3 | No | Yes | Normal | Lung, Peripheral neuropathy | Normal | IV GC 40 mg twice a day for 1 week,  IV CYC 0.8 g once,  IVIG 20 g daily for 5 days | | 33 |
| 52 | 21 | F | c-ANCA | No | Yes | Hypogonadism | ENT, CNS, Eyes, Joints | Large sellar mass with  central hypointensity and peripheral hyperintensity | GC+CYC | | 34 |
| 53 | 29 | M | c-ANCA and PR3 | No | No | Hypothyroidism,  Adrenal insufficiency,  Hypogonadism,  GH and IGF-1 deficiency | ENT, CNS (cerebral vasculitis) | Cystic sellar lesion | GC+CYC | | 35 |
| 54 | 26 | M | Not reported | No | Yes | Normal | ENT, Kidney, Lung, Eyes | Abnormal enhancement of the pituitary | GC, oral CYC, IVIG | | 36 |
| 55 | 21 | F | c-ANCA and PR3 | No | Yes | Hypothyroidism,  Adrenal insufficiency, Hypogonadism | ENT, Lung | Enlarged pituitary and infundibulum, heterogeneous enhancement | GC+IV CYC | | 37 |
| 56 | 19 | M | c-ANCA and PR3 | No | Yes | Normal | ENT, Kidney, Lung, Skin,  Eyes | Enlargement of pituitary gland, dural enhancement and infiltration | GC, CYC,  MTX 25 mg, INF 5 mg/kg,  MMF 2 g, Rituximab 1 g | | 38 |
| 57 | 33 | F | c-ANCA | No | Yes | Prolactin and thyroid function were normal. Pituitary assessment was hampered by her  corticosteroid therapy and  the oral contraceptive pill. | ENT | Enlarged gland containing a poorly enhancing lesion with suprasellar extension, loss of posterior signal | GC, oral CYC, AZA, MMF 2 g, Rituximab 1 g | | 38 |
| 58 | 26 | M | c-ANCA | No | Yes | Hypothyroidism | ENT, Lung | Enlarged pituitary and  thickened stalk | GC, IV CYC, Alemtuzumab, MMF; Rituximab | | 38 |
| 59 | 22 | F | c-ANCA and PR3 | No | Yes | Hypothyroidism,  Adrenal insufficiency, Hypogonadism | ENT, CNS, Skin, Eyes, Gut | Heterogeneous enhancing pituitary mass,  expansion into right cavernous sinus, enhancement of meninges bilaterally | GC+CYC | | 39 |
| 60 | 52 | M | MPO-ANCA | Yes | Yes | Not reported | ENT, Eyes | Hypophysitis | GC+IV CYC | | 40 |
| 61 | 47 | M | c-ANCA and PR3 | No | Yes | Normal | ENT, Kidney, Skin | Enlarged heterogeneous pituitary gland | GC+CYC | 41 | |
| 62 | 55 | F | c-ANCA and PR3 | Yes | Yes | Mild hyperprolactinemia | ENT, Kidney, Lung, peripheral neuropathy, Joints | Loss of posterior signal, stalk thickened | GC, CYC, Rituximab | 41 | |
| 63 | 47 | F | p-ANCA and MPO | Yes | Yes | Mild hyperprolactinemia | ENT, CNS, Lung, Joints | Enlarged heterogeneous pituitary gland, stalk thickened | GC+MTX | 41 | |
| 64 | 54 | F | Not reported | Yes | Yes | hypogonadism | ENT, Lung, Skin, CNS, Peripheral neuropathy | Sellar mass of granulomatous characteristic | GC+CYC | 41 | |
| 65 | middle-aged | F | c-ANCA and PR3 | NO | No | hypothyroidism and hypogonadism | Normal | Sellar mass | CYC+AZA | 42 | |
| 66 | 16 | F | c-ANCA | Yes | Yes | Hypothyroidism,  Adrenal insufficiency,  Hypogonadism,  GH and IGF-1 deficiency | Joints | Sellar mass, stalk thickened, loss of posterior signal | GC+CYC | 43 | |
| M=male, F=Female, DI=diabetes insipidus, c-ANCA=cytoplasmic perinuclear anti-neutrophil cytoplasmic antibody, MPO=myeloperoxidase, PR3= proteinase 3, ENT=ear, nose, and throat, CNS=central nervous system, GH=growth hormone, IGF-1=insulin-like growth factor-1, GC=glucocorticoid, CYC= cyclophosphamide, AZA =azathioprine, INF=infliximab, IVIG=intravenous immunoglobulin, MMF=mycophenolate mofetil, MTX=methotrexate, IV=intravenous. | | | | | | | | | | | |
