# Supplementary material for: Pituitary Involvement in Granulomatosis with Polyangiitis: A Retrospective Analysis in a Single Chinese Hospital and a Literature Review
Source: Int J Endocrinol. 2019 Nov 6;2019:2176878. doi: 10.1155/2019/2176878 (PMC6874975; doi:10.1155/2019/2176878)
Supplement: Supplementary Materials — Supplementary Table 1: characteristics, ANCA results, pituitary function, radiographic findings, treatment, and outcome of patients with GPA-related pituitary disease. Supplementary Table 2: raw follow-up of patients with GPA-related pituitary disease. [file 2176878.f1.zip › 2176878.f1/Supplementary Table 2.docx]

| **Supplementary Table 2. Raw Follow-Up of Patients with GPA-Related Pituitary Disease** | | | | |
| --- | --- | --- | --- | --- |
| **Case**  **No** | **Pituitary imaging** | **Anterior pituitary function** | **Posterior pituitary**  **function** | **Systemic**  **disease** |
| 1 | No change | Remission | _ | Remission |
| 2 | No change | Persistent insufficiency | Remission | Remission |
| 3 | No change | Partial remission | Remission | Not reported |
| 4 | Normalized | Persistent insufficiency | Remission | Remission |
| 5 | Reduction, persistent  loss of posterior signal | Remission | Remission | Remission |
| 6 | Normalized | Persistent insufficiency | Persistent insufficiency | Relapse |
| 7 | Reduction | Persistent insufficiency | _ | Remission |
| 8 | Reduction | _ | Partial remission | Remission |
| 9 | Not reported | _ | Persistent insufficiency | Remission |
| 10 | Normalized | Persistent insufficiency | _ | Relapse |
| 11 | Reduction | Persistent insufficiency | Persistent insufficiency | Stabilized |
| 12 | No change, persistent  loss of posterior signal | Persistent insufficiency | Persistent insufficiency | Remission |
| 13 | Not reported | Persistent insufficiency | _ | Remission |
| 14 | Not reported | Persistent insufficiency | Persistent insufficiency | Remission |
| 15 | Normalized | Partial remission | Persistent insufficiency | Remission |
| 16 | Normalized | Persistent insufficiency | Persistent insufficiency | Remission |
| 17 | No change | Persistent insufficiency | Remission | Remission |
| 18 | Reduction | Persistent insufficiency | Persistent insufficiency | Remission |
| 19 | Not reported | _ | Remission | Remission |
| 20 | Reduction | Persistent insufficiency | Persistent insufficiency | Not reported |
| 21 | Normalized, persistent  loss of posterior signal | _ | Persistent insufficiency | Remission |
| 22 | Reduction | _ | Persistent insufficiency | Remission |
| 23 | Normalized | _ | Persistent insufficiency | Remission |
| 24 | Reduction, persistent  loss of posterior signal | Persistent insufficiency | Persistent insufficiency | Not reported |
| 25 | No change | Persistent insufficiency | Partial remission (desmopressin was gradually discontinued, with both a mild increment in thirst and daily diuresis) | Not reported |
| 26 | Not reported | _ | Not reported | Remission |
| 27 | Reduction | Persistent insufficiency | _ | Remission |
| 28 | Not reported | Remission | Persistent insufficiency | Remission |
| 29 | Not reported | _ | Persistent insufficiency | Relapse then remission |
| 30 | Not reported | _ | Remission | Relapse then remission |
| 31 | Not reported | _ | Persistent insufficiency | Remission |
| 32 | Normalized | _ | Persistent insufficiency | Not reported |
| 33 | Normalized | Persistent insufficiency | Persistent insufficiency | Remission |
| 34 | Reduction | Remission | Persistent insufficiency | Remission |
| 35 | Not reported | _ | Remission | Remission |
| 36 | No change | Persistent insufficiency | Persistent insufficiency | Relapse then remission |
| 37 | No change | Persistent insufficiency | Persistent insufficiency | Stabilized |
| 38 | Nearly normalized | Not reported | Not reported | Remission |
| 39 | Not reported | Persistent insufficiency | Persistent insufficiency | Remission |
| 40 | Normalized, persistent  loss of posterior signal | Persistent insufficiency | Persistent insufficiency | Relapse then remission |
| 41 | Normalized, persistent  loss of posterior signal | Not reported | Not reported | Relapse then  stabilized |
| 42 | Normalized | Not reported | Remission | Relapse then  stabilized |
| 43 | Not reported | _ | Persistent insufficiency | Remission |
| 44 | No change | Remission | _ | Relapse then  stabilized |
| 45 | Not reported | _ | Remission | Relapse then  stabilized |
| 46 | Normalized | Not reported | Not reported | Remission |
| 47 | Reduction | _ | Not reported | Relapse then  remission |
| 48 | Reduction | Persistent insufficiency | Persistent insufficiency | Relapse then  stabilized |
| 49 | Reduction | Remission | _ | Remission |
| 50 | Normalized, persistent  loss of posterior signal | Not reported | Persistent insufficiency | Remission |
| 51 | Not reported | _ | Remission | Remission |
| 52 | Not reported | Remission | Remission | Remission |
| 53 | Reduction | Not reported | _ | Remission |
| 54 | Death | _ | Death | Death |
| 55 | Not reported | Not reported | Not reported | Relapse then  stabilized |
| 56 | Normalized | _ | Persistent insufficiency | Remission |
| 57 | Reduction | Not reported | Not reported | Relapse then  remission |
| 58 | Further enlargement | Not reported | Not reported | Progression |
| 59 | Death | Death | Death | Death |
| 60 | Reduction | _ | Remission | Not reported |
| 61 | No change | _ | Persistent insufficiency | Remission |
| 62 | Normalized | Remission | Persistent insufficiency | Remission |
| 63 | No change， persistent  loss of posterior signal | Not reported | Persistent insufficiency | Remission |
| 64 | Reduction | Not reported | Persistent insufficiency | Remission |
| 65 | Not reported | Not reported | _ | Not reported |
| 66 | Reduction | Persistent insufficiency | Persistent insufficiency | Not reported |
| _ = not reported or normal at the onset of the disease | | | |  |
